# Mycobacterial Infection of the Gallbladder Masquerading as Gallbladder Cancer with a False Positive Pet Scan

**DOI:** 10.1155/2013/828631

**Published:** 2013-03-17

**Authors:** Adeeb Majid, Ravish Sanghi Raju, Markus Trochsler, Harsh A. Kanhere, Guy J. Maddern

**Affiliations:** ^1^The Queen Elizabeth Hospital, Division of Surgery, Level 6A, 28 Woodville Road, Woodville South, SA 5011, Australia; ^2^Queen Elizabeth Hospital, Discipline of Surgery, University of Adelaide, Woodville, SA 5011, Australia

## Abstract

Isolated mycobacterial infection of gall bladder is an extremely rare entity. Only anecdotal reports are evident in the literature. A preoperative diagnosis of mycobacterial infection of gallbladder is therefore very difficult. The case of a 72-year-old male who underwent surgery for suspected gallbladder cancer is presented. The diagnosis of cancer was based on radiological findings and an abnormal uptake of fluorine-18-fluoro-2-deoxy-D-glucose (FDG) on positron emission tomography (PET) scan whilst being followed up for colorectal cancer. He underwent cholecystectomy and gallbladder bed resection. Histopathology was consistent with mycobacterial infection of the gallbladder.

## 1. Introduction

Primary mycobacterial infection of the gallbladder is rare and very few cases have been reported in the literature. The first case was reported in 1870 by Gaucher [1].

Gallbladder tuberculosis (GT) can mimic gallbladder carcinoma in a patient presenting with gallbladder mass [2, 3]. Due to lack of pathognomic features on radiology, it is often diagnosed only after histopathology of resected specimen [4].

## 2. Case Report

A 72-year-old Vietnamese man living in Australia for 30 years was found to have a gallbladder mass on imaging whilst on followup for Dukes B rectal cancer. He had undergone a low anterior resection in 2004. He did not have past history of tuberculosis.

**Figure 1:**
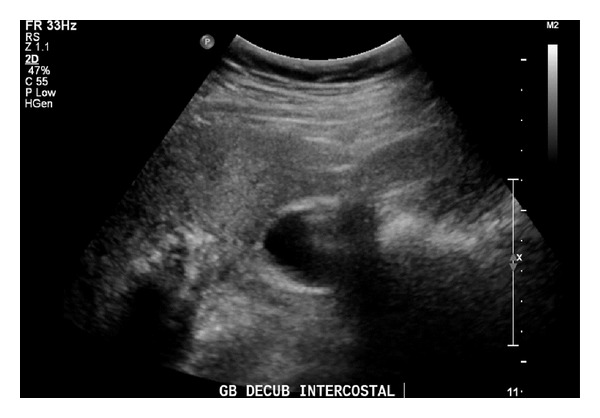
Abnormal gallbladder with thickened wall and mass in the fundus on ultrasound.

**Figure 2:**
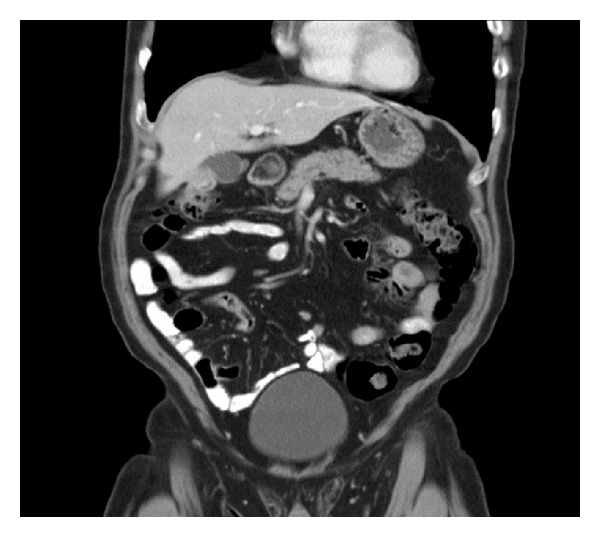
CT scan showing ovoid mass in gallbladder.

Ultrasound (US) abdomen revealed an abnormal gallbladder with thickened wall and solid mass at the fundus raising the possibility of neoplasia (Figure 1). This was corroborated by a computed tomography (CT) scan which showed a 3 cm diameter irregularly ovoid shaped enhancing mass in the gallbladder fossa. The mass appeared to protrude into the gall bladder lumen and was abutting the superior aspect of hepatic flexure suggestive of an origin either from the gallbladder or hepatic flexure of the colon. There was no evidence of enlarged intra-abdominal nodes or calcification (Figure 2).

**Figure 3:**
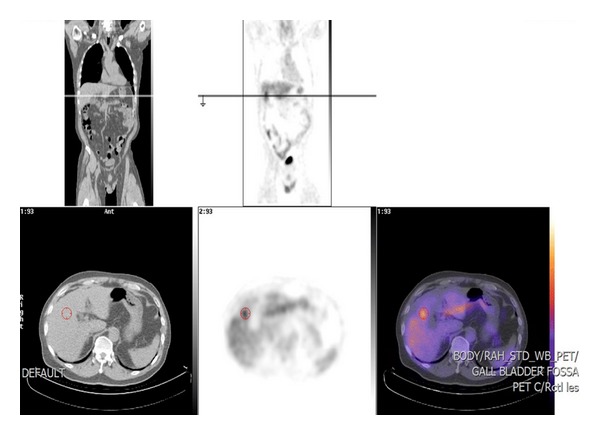
High FDG uptake in gallbladder fossa on PET scan.

Colonoscopy did not reveal any intraluminal abnormality. PET scan revealed a focus of prominent FDG accumulation in the vicinity of the gall bladder mass lesion raising a suspicion for malignancy (Figure 3).

These findings were discussed in a multidisciplinary meeting. The consensus opinion was to perform an open cholecystectomy and gallbladder bed resection in view of the likelihood of malignancy. Intraoperative findings revealed a 50 × 35 × 35 mm gallbladder with a hard nodular mass in the fundus. This was firmly attached to the hepatic flexure. Cholecystectomy with resection of gallbladder bed was performed. The specimen was sent for frozen section which revealed necrotising granuloma suggestive of mycobacterial infection with no evidence of malignancy. This was confirmed on paraffin section.

The postoperative course was uneventful. The tissue was sent for polymerase chain reaction (PCR) test which confirmed the diagnosis. Infectious diseases consultation was obtained. The patient was commenced on a course of antitubercular therapy with isoniazid, rifampicin, pyrazinamide, and ethambutol for six months. He has recovered well from the surgery and is making good progress on antitubercular treatment.

## 3. Discussion

Isolated mycobacterial infection of gallbladder is an extremely rare entity especially in the western world. Most cases of GT have been reported in the Asian literature [2–5].

The gallbladder is highly resistant to tubercular infection possibly due to the inhibitory function of bile [2, 3]. The route of infection may be peritoneal, hematogenous, or lymphatic [3]. The differential diagnosis of GT includes acute and chronic cholecystitis, polypoid lesions, and gallbladder carcinoma [3].

The presence of a mass that fills the gallbladder associated with cholelithiasis is indistinguishable from carcinoma of the gallbladder [2]. Moreover, both gallbladder tuberculosis and carcinoma can lead to regional lymphadenopathy. The presence of liver metastasis or liver infiltration suggests the presence of gallbladder carcinoma. On the other hand, lung lesions or mesenteric thickening is frequent in patients with tubercular infection [6].

Ultrasound and CT scan may show an enlarged gallbladder, irregularly thickened gallbladder wall, or a soft tissue mass with necrosis or calcification, or nodular lesions, although neither ultrasound nor CT lesions are highly specific [5].

A preoperative diagnosis is difficult to obtain due to the lack of pathognomic features on radiology. GT is therefore mostly diagnosed only after histopathology of the resected specimen [4].

Positron-emission tomography (PET) is a noninvasive method to assess metabolism in vivo by means of positron-emitting radiolabeled tracers. Fluorine-18-fluoro-2-deoxy-D-glucose (FDG) is a glucose analogue that is phosphorylated in the cells but not further metabolized.

Most malignant tumors show increased uptake of FDG. Malignant transformation and growth of tumor cells is associated with overexpression of glucose transporters and increased hexokinase activity [7].

PET scans have sensitivity of 0.80 and specificity of 0.82 in the diagnosis of gallbladder cancer [8]. A study by Rodríguez-Fernández et al. [9] corroborating these figures found that FDG-PET provided reliable diagnosis of gallbladder cancer with 75% sensitivity, 87.5% specificity, and 81.3% accuracy.

To our knowledge this is only the second paper documenting an FDG avid mass in the gallbladder being a mycobacterial infection [6]. Most previous papers describe gallbladder involvement as a part of generalised abdominal or extra-abdominal disease [2, 3, 5]. Isolated gallbladder affliction was a distinct feature in this case. Whilst the commonest presentation of GT is with chronic cholecystitis, it can also present as a mass lesion in the gallbladder, which is difficult to distinguish from a gallbladder cancer, or with a cholecystoenteric fistula [6].

In a culturally diverse country such as Australia, many patients from Asian and south east Asian backgrounds are treated. Mycobacterial infections are endemic in these regions. One should consider a differential diagnosis of gallbladder mycobacterial infection when treating a patient with gallbladder mass in this scenario.
